# Solvent-Free Mechanochemical Synthesis and Characterization of Nickel Tellurides with Various Stoichiometries: NiTe, NiTe_2_ and Ni_2_Te_3_

**DOI:** 10.3390/nano11081959

**Published:** 2021-07-29

**Authors:** Matjaž Kristl, Sašo Gyergyek, Srečo D. Škapin, Janja Kristl

**Affiliations:** 1 Faculty of Chemistry and Chemical Engineering, University of Maribor, 2000 Maribor, Slovenia; saso.gyergyek@ijs.si; 2 Synthesis of Materials Department K8, Jožef Stefan Institute, 1000 Ljubljana, Slovenia; 3 Advanced Materials Department K9, Jožef Stefan Institute, 1000 Ljubljana, Slovenia; sreco.skapin@ijs.si; 4 Faculty of Agriculture and Life Sciences, University of Maribor, 2000 Maribor, Slovenia; janja.kristl@um.si

**Keywords:** nickel, telluride, nanoparticles, magnetic properties, band gap, mechanochemistry

## Abstract

The paper reports the synthesis of nickel tellurides via a mechanochemical method from elemental precursors. NiTe, NiTe_2_, and Ni_2_Te_3_ were prepared by milling in stainless steel vials under nitrogen, using milling times from 1 h to 12 h. The products were characterized by powder X-ray diffraction (pXRD), scanning electron microscopy (SEM), transmission electron microscopy (TEM), energy-dispersive X-ray spectroscopy (EDX), dynamic light scattering (DLS), vibrating sample magnetometer (VSM), UV-VIS spectrometry, and thermal analysis (TGA and DSC). The products were obtained in the form of aggregates, several hundreds of nanometers in size, consisting of smaller nanosized crystallites. The magnetic measurements revealed a ferromagnetic behavior at room temperature. The band gap energies calculated using Tauc plots for NiTe, NiTe_2_, and Ni_2_Te_3_ were 3.59, 3.94, and 3.70 eV, respectively. The mechanochemical process has proved to be a simple and successful method for the preparation of binary nickel tellurides, avoiding the use of solvents, toxic precursors, and energy-consuming reaction conditions.

## 1. Introduction

Transition metal chalcogenides have gained much attention over the past two decades in the field of material science. Among them, nickel chalcogenides have attracted significant interest due to their various phases and stoichiometries, exhibiting a wide range of physical and chemical properties [1]. Most of the earlier studies report on research in the Ni-S [2,3] and, to a lesser extent, Ni-Se systems, while nickel tellurides seem to be less intensively studied. Nickel and tellurium are known to form a continuous series of compounds between the compositions of NiTe and NiTe_2_ [4,5]. All stoichiometric nickel tellurides crystallize in a hexagonal structure, with NiTe having a NiAs (B8) type of structure, while NiTe_2_ has a Cd(OH)_2_(C6) type of structure. Ni_2_Te_3_ also crystallizes in a hexagonal structure with crystal parameters between those of NiTe and NiTe_2_ [6].

Recently, there have been many studies reporting the potential applications of nickel tellurides in various cutting-edge research areas. Nickel tellurides have been tested as electrode material for the determination of uric acid and adenine in physiological fluids [7], glucose sensors [8], anode material for Li-ion batteries [9], supercapacitor [10,11,12], and solar cell [13] materials. The most attractive research area seems to be the possible use of nickel tellurides as electrocatalysts for hydrogen/oxygen evolution [14,15,16,17,18,19,20]. The lower electronegativity of Te, when compared to S and Se, provides tellurides with a more metallic character, giving them ferromagnetic behavior and properties, which outperform sulfides and selenides when applied as electrocatalysts [21].

Traditionally, nickel tellurides have been prepared by the solid-state reaction, requiring elevated temperatures, inert atmosphere or evacuated quartz ampules, and long reaction times reaching up to 10 days [14,22,23], making them inconvenient for the preparation of nanosized products [7]. Different alternative approaches have been proposed to overcome this problem, like the precipitation of tellurides from aqueous solutions using unstable and very toxic gaseous H_2_Te [1], solid-state metathesis reaction [24], and the molecular precursor method [25]. More recently, NiTe and NiTe_2_ thin films have been prepared by electrodeposition [26] and chemical vapor deposition [27]. The hydro/solvothermal method is also widely used for preparation of nickel tellurides with different stoichiometries like NiTe [4,9,10,11,12,19,28,29], NiTe_2_ [1,20,30,31], NiTe/NiTe_2_ mixed phases [15], Ni_3_Te_2_ [8] as well as a whole series of NiTe*_x_* nanorods with 1 ≤ *x* ≤ 2 [6].

The mechanochemical method, also known as mechanical alloying, is a convenient solid-state processing technique in which the precursor powders are exposed to repeated welding, fracturing, and re-welding in a high energy ball mill. The method has been developed in the 1970s for the fabrication of nickel- and iron-base superalloys for applications in the aerospace industry [32,33] and is nowadays widely used in the processing of advanced materials like alloys [34], bimetallic nanocatalysts [35], oxide materials [36,37], carbon-based materials [38,39,40], etc. The first reports of mechanochemical synthesis of metal chalcogenides date back to the 1990s, and since then, a number of nanosized sulfides, selenides, and tellurides have been prepared using this method. Our group earlier reported on the mechanochemical synthesis of copper and cadmium chalcogenides (CuS, CuSe_2_, Cu_7_Te_5_, CdS, CdSe, and CdTe) [41] as well as mechanochemical preparation of Al_2_S_3_, Al_2_Se_3_, Ga_2_S_3_, and Ga_2_Se_3_ [42]. More recently, syntheses of copper-nickel antimony sulfide [43]; lead bismuth telluride [44]; zinc sulfide [45]; and silver, indium, and gallium selenides [46] using the mechanochemical approach have been reported. After a thorough search, we found only one paper reported the preparation of nickel tellurides by mechanical alloying: Campos prepared NiTe nanocrystals by milling a stoichiometric mixture of elemental Ni and Te powders in an argon atmosphere for 3 h–10 h [47]. The main aim of our research was to study mechanochemical syntheses in the Ni:Te system using changing molar ratios with the goal to prepare different members of the continuous NiTe*_x_* series (1 ≤ *x* ≤ 2). In this paper, the preparation of three nickel tellurides with the compositions NiTe, NiTe_2_, and Ni_2_Te_3_ by a solventless and facile mechanochemical method is reported.

## 2. Materials and Methods

A SPEX 8000 M ball mill (SPEX SamplePrep, Metuchen, NJ, USA), with a 65 mL stainless steel vial and two 12.7 mm stainless steel grinding balls were used for the synthesis. The precursors Ni (powder, ~3 micron, 99.7%, Sigma-Aldrich, Sant Louis, MO, USA) and Te (powder, ~200 mesh, 99.8%, Sigma-Aldrich, Sant Louis, MO, USA) were used as supplied, without further purification. Ni:Te mixtures in molar ratios 1:1, 1:2, and 2:3 were sealed together with the balls in the vial inside a glove box under nitrogen. The ball-to-powder mass ratio was 10:1 during all experiments. For the 1:1 sample, 0.473 g (=0.008 mol) of Ni and 1.027 g (=0.008 mol) of Te were used. The samples were mechanically alloyed, applying milling times ranging from 1 h to 12 h. Powder diffraction (pXRD) data were collected on a D5005 diffractometer, (Bruker-Siemens, Hamburg, Germany) using copper radiation (*λ* = 1.54178 Å, 30 kV, 40 mA), scanning range from 10° to 70°, step size 0.02° and time/step = 1 s. The morphological characterization was performed using scanning electron microscope HRSEM Sirion NC400 FEI (Thermo Fisher Scientific, Waltham, MA, USA) coupled to energy dispersive spectroscopy (EDS) detector. Transmission electron microscopy (TEM) measurements were performed using a JEOL 2100 microscope (JEOL Inc., Peabody, MA, USA). The obtained powders were dispersed in ethanol using an ultrasonic bath and placed on a carbon-coated copper grid. The elemental composition of the particles was analyzed using a Jeol JED 2300 EDXS system, (JEOL Inc., Peabody, MA, USA). The hydrodynamic particle size was measured by dynamic light scattering (DLS) with a Zetasizer Nano-S equipment (Malvern Instruments, Malvern, UK) using disposable 1 cm PS cuvettes. The magnetic properties were investigated with a vibrating sample magnetometer (VSM) Lakeshore 7307 (Lake Shore Cryotronics, Westerville, OH, USA). A UV-VIS spectrometer Varian Cary 50 Bio (Varian Inc., Palo Alto, CA, USA) was used to study the optical properties of the prepared nickel tellurides. The samples were dispersed in ethanol in an ultrasonic bath prior to the measurement. The nickel content was measured on a Varian SpectrAA-10 flame atomic spectrometer (AAS) (Varian Inc., Palo Alto, CA, USA) after digestion of the samples with concentrated HNO_3_ in PTFE vessels in a microwave oven (MDS-2000, CEM Corporation, Matthews, NC, USA). Thermal analysis of the samples was carried out in the Mettler TGA/SDTA 851e system (Mettler Toledo, Switzerland) using 70 µL alumina crucibles (TGA) and Mettler DSC 20 (Mettler Toledo, Switzerland) using 40 µL aluminum crucibles (DSC). The heating rate was 10 K/min while the temperature range was from 30 to 900 °C (TGA) and from 30 to 600 °C (DSC).

## 3. Results and Discussion

**Figure 1:**
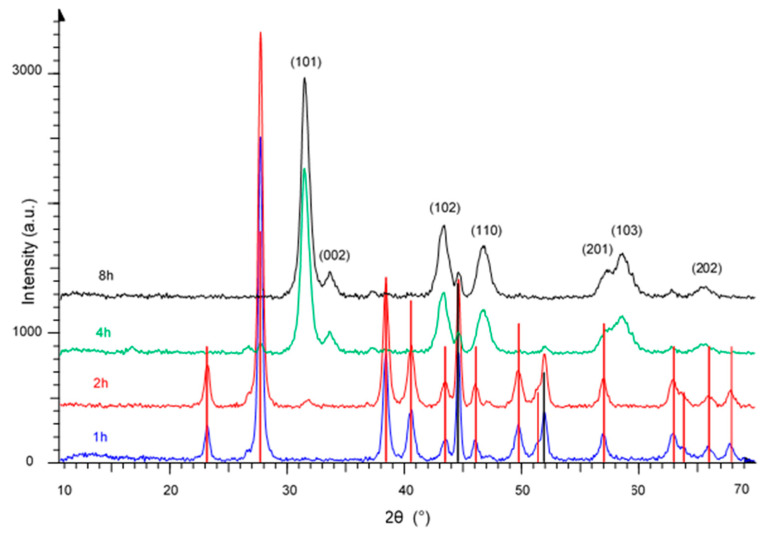
XRD patterns of NiTe prepared mechanochemically after 1 h, 2 h, 4 h, and 8 h milling time. Black and red vertical lines represent the precursors Ni and Te, respectively.

The diffraction patterns of the products, obtained mechanochemically from a Ni:Te = 1:1 mixture using 1 h, 2 h, 4 h, and 8 h milling time, are shown in Figure 1. After 1 h milling time, all the peaks still belong to both precursors, i.e., nickel (marked by black vertical lines, JCPDS file No. 00-004-0850) and tellurium (marked by red vertical lines, JCPDS file No. 00-004-0554). First visible peaks belonging to nickel telluride were observed after 2 h milling time. After 4 h, all major peaks could be identified as belonging to hexagonal NiTe (JCPDS file No. 00-038-1393), while after 8 h, the product was obtained without detectable impurities. The crystallite size was calculated from the Scherrer equation:

$$d_{x}=\frac{0.94\cdot \lambda \cdot 57.3}{\beta \cdot \cos\theta }$$

Here, *λ* is the wavelength of the X-ray radiation (nm), *β* the full width at half maximum (FWHM) of the corresponding peak (^o^), and *θ* is the diffraction angle (^o^). The crystallite size calculated from the average of (101) and (110) peaks was found to be 12 nm.

**Figure 2:**
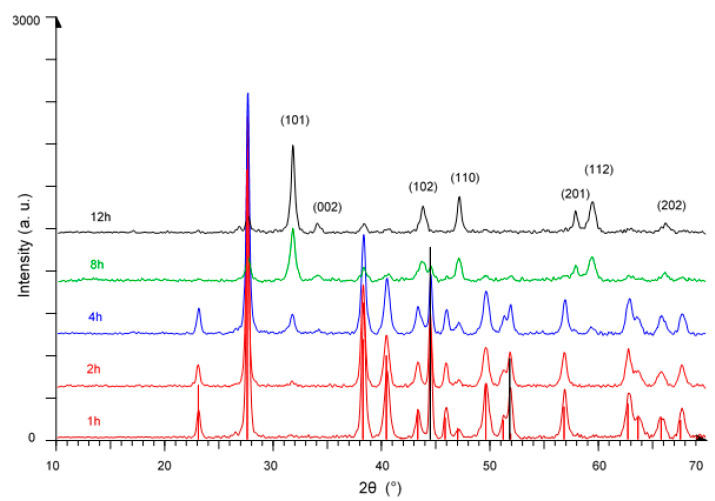
XRD patterns of NiTe_2_ prepared mechanochemically after 1 h, 2 h, 4 h, 8 h, and 12 h milling time. Black and red vertical lines represent the precursors Ni and Te, respectively.

Figure 2 shows diffraction patterns of products obtained by milling a Ni:Te mixture in the molar ratio of 1:2. When compared to Figure 1, one major difference is the longer milling time needed for the formation of the product. After 4 h milling time, the predominant peaks are still those of both precursors, Ni and Te, next to nickel telluride which starts to form. Even after 8 h, unreacted Ni and Te are clearly visible. By 12 h milling time, we obtained NiTe_2_ (JCPDS file No. 00-008-0004) with an average crystallite size of 23 nm.

**Figure 3:**
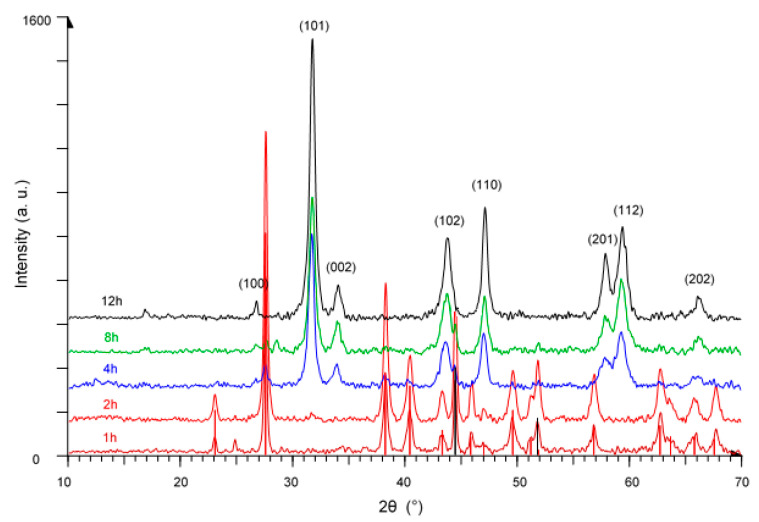
XRD patterns of Ni_2_Te_3_ prepared mechanochemically after 1 h, 2 h, 4 h, 8 h, and 12 h milling time. Black and red vertical lines represent the precursors Ni and Te, respectively.

The diffraction patterns of products obtained by milling a Ni:Te mixture in the molar ratio of 2:3 are presented in Figure 3. Like in the previous cases, the patterns after 1 h and 2 h show predominantly peaks of nickel and tellurium, while nickel telluride Ni_2_Te_3_, JCPDS file No. 00-002-0810, starts to emerge after 4 h milling time. After 12 h milling time, only peaks of Ni_2_Te_3_ with an average crystallite size of 18 nm are visible.

Attempts to prepare Ni-rich stoichiometric compounds by milling a Ni:Te mixture in the molar ratio of 2:1 did not yield the desired product. XRD patterns of as-prepared powders showed a mixture of NiTe and unreacted Ni.

**Figure 4:**
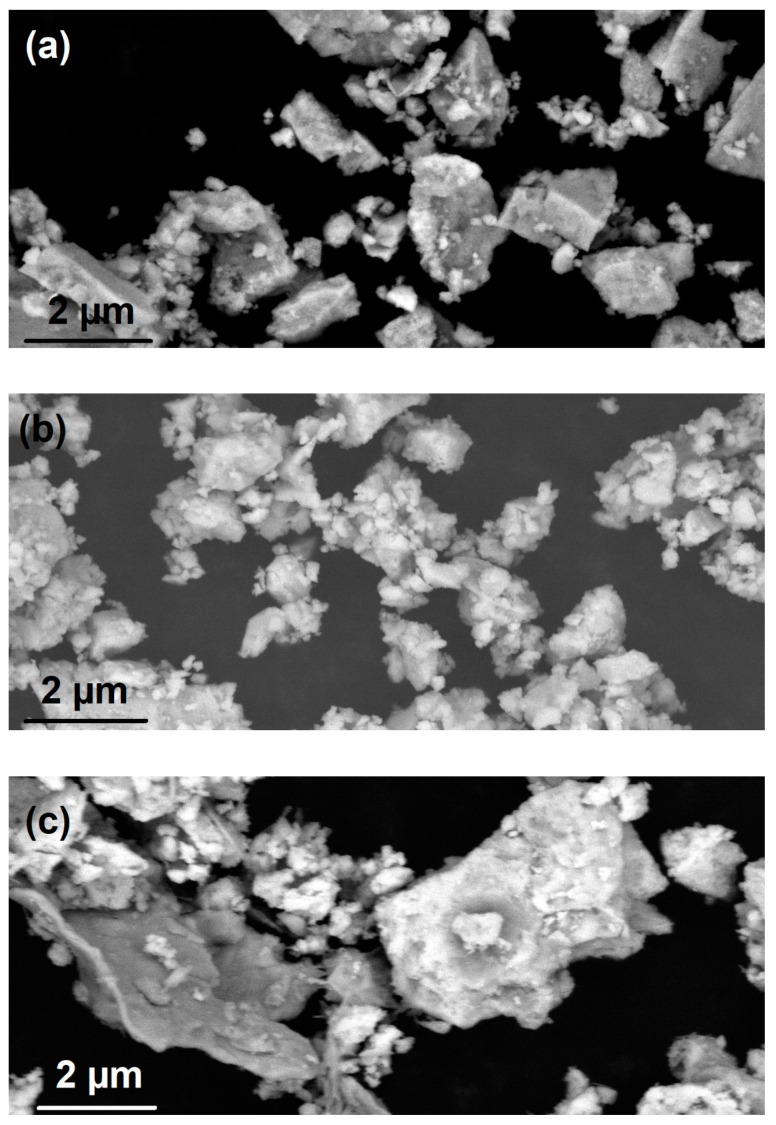

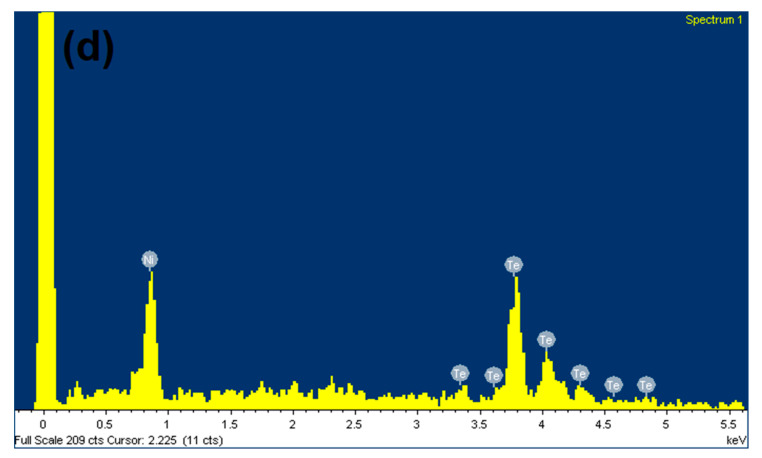
SEM images of (**a**) NiTe after 8 h milling time, (**b**) NiTe_2_ after 12 h milling time, and (**c**) Ni_2_Te_3_ after 12 h milling time. Figure 4 (**d**) represents the compositional analysis of the NiTe sample.

The surface morphology of the samples has been studied by scanning electron microscopy. The deposited samples were observed under an acceleration voltage of 10 kV. SEM images of NiTe, NiTe_2_, and Ni_2_Te_3_, are shown in Figure 4a–c. The samples are formed irregularly shaped agglomerates ranging from several hundreds of nm to micron-sized clusters. Most of the agglomerates are formed from smaller rounded particles, which is characteristic of powders prepared by dry mechanochemical reactions [37], while some clear straight edges of freshly crushed grains can also be observed, especially in Figure 4a. The results of EDX analysis (typical pattern shown in Figure 4d) confirm the presence of Ni and Te in the sample and the absence of impurities.

**Figure 5:**
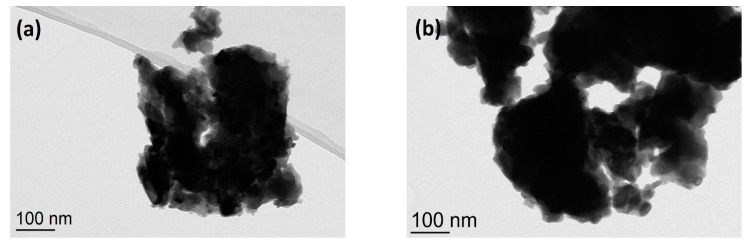

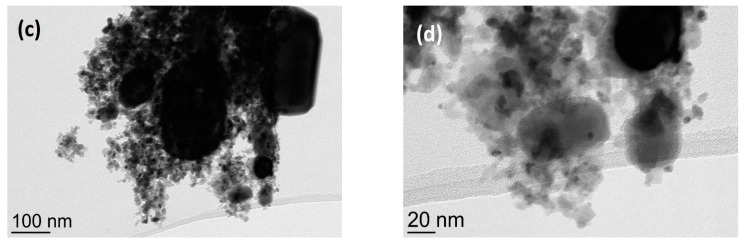
TEM images of (**a**) NiTe after 8 h milling time, (**b**) NiTe_2_ after 12 h milling time, and (**c**,**d**) Ni_2_Te_3_ after 12 h milling time.

TEM images of NiTe (a), NiTe_2_ (b), and Ni_2_Te_3_ (c) nanoparticles, obtained by mechanical milling after 8 h, 12 h, and 12 h, respectively, are shown in Figure 5a–c. The products are composed of irregularly shaped clusters reaching up to several hundred nanometers, which can be explained with huge mechanical forces during the milling process. However, at higher magnifications, smaller primary spherical nanoparticles with diameters in the 10–20 nm range can be observed, as can be seen in Figure 5d. The EDX analysis showed the presence of Ni and Te in the ratios, which correspond fairly well to results obtained from pXRD analysis: 48 at.% Ni + 52 at.% Te for NiTe, 29 at.% Ni + 71 at.% Te for NiTe_2_, and 39 at.% Ni + 61 at.% Te for Ni_2_Te_3_. The results are in good agreement with Ni content determined by AAS with measured values of 30.9 wt.% Ni for NiTe (calc. 31.5%), 18.3 wt.% Ni for NiTe_2_ (calc. 18.7%), and 23.3 wt.% Ni for Ni_2_Te_3_ (calc. 23.5%). No detectable amount of nickel oxides could be detected neither by EDX nor by powder XRD analysis. It is worthwhile to mention that during initial experiments, when Ni and Te were loaded into the milling vial under ambient air, NiO was always observed by EDX and XRD next to prevailing nickel tellurides.

**Figure 6:**
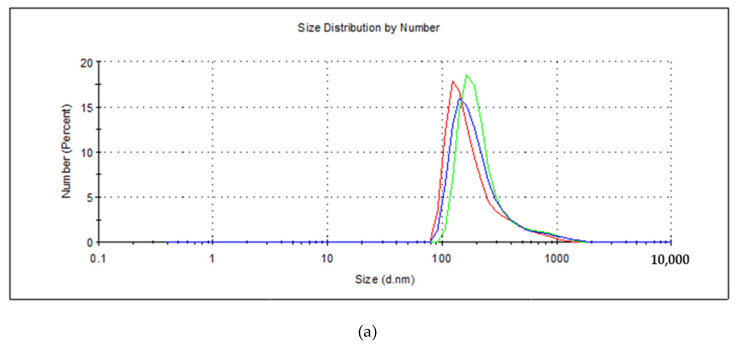

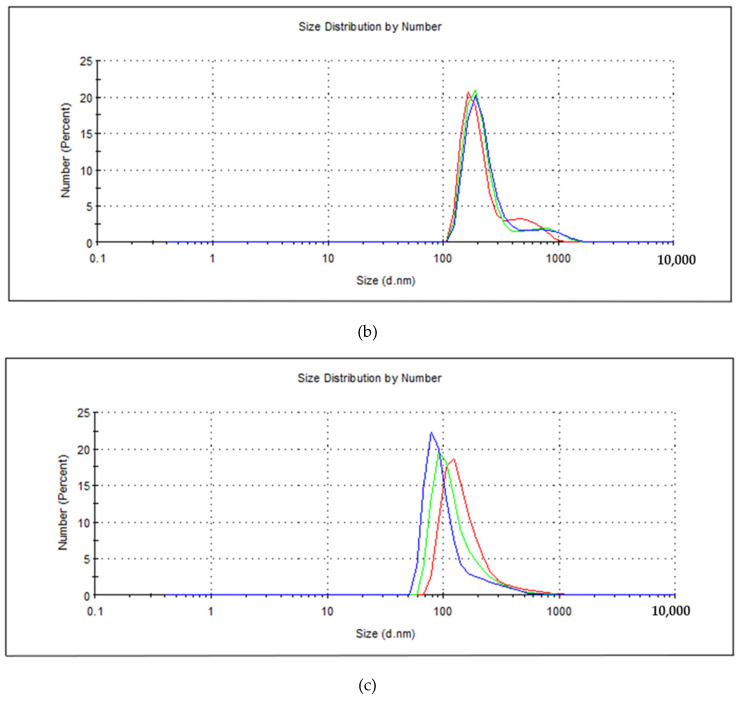
Results of DLS measurements for the number of particles in % of mechanochemically prepared NiTe (**a**), NiTe_2_ (**b**) and Ni_2_Te_3_ (**c**).

To further confirm the morphology of the as-obtained nickel tellurides, particle size and hydrodynamic size distribution were measured by DLS in water after dispersion in an ultrasonic bath. Three independent measurements were performed for each of the samples and averaged for particle size calculations, yielding values of 204, 221, and 115 nm for NiTe, NiTe_2_, and Ni_2_Te_3_, as shown in Figure 6. It must be noted that results obtained by DLS generally tend to give higher values than those obtained by electron microscopy due to the fact that in the former case, particles are surrounded by water molecules [48,49,50].

**Figure 7:**
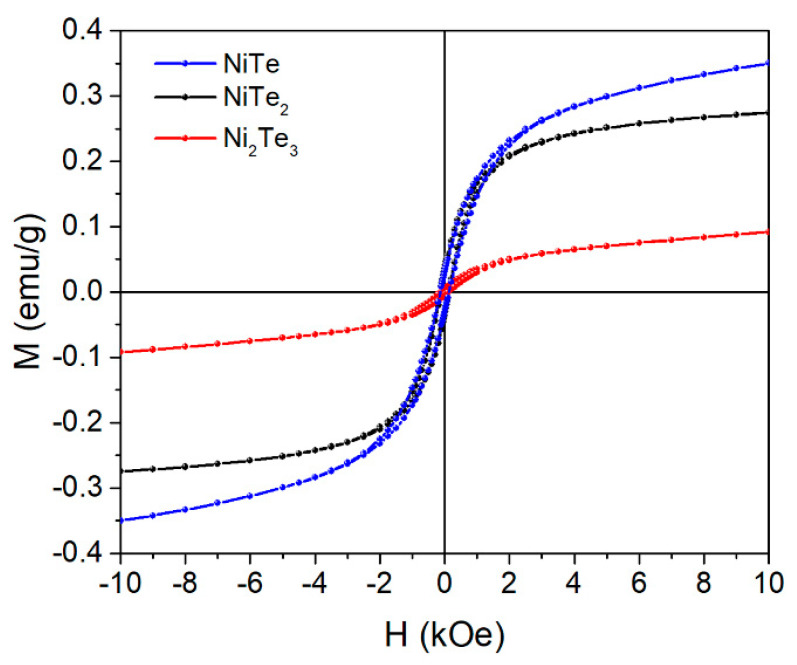
Room-temperature magnetization curves for NiTe after 8 h milling time, NiTe_2_ after 12 h milling time, and Ni_2_Te_3_ after 12 h milling time. The line serves as a guide to the eye.

The literature data reporting on the magnetic behavior of nickel tellurides are scarce and seem to be inconsistent. While Zhang et al. state that NiTe is an antiferromagnetic compound [4], Lei et al. report paramagnetic behavior of hydrothermally prepared NiTe nanorods [6], which seems to be in accordance with bulk NiTe prepared by the solid-state reaction in an evacuated quartz ampoule [23]. On the contrary, Wan et al. reported the ferromagnetic properties of hydrothermally prepared nanowires [29], which might be attributed to the presence of small amounts of ferromagnetic impurities [6]. The magnetism of NiTe*_x_* nanoparticles (1 ≤ *x* ≤ 2) is even less researched. The magnetic moment of Ni atoms is ‘diluted’ by tellurium atoms; thus, NiTe_2_ shows diamagnetic properties at 100 K. NiTe*_x_*nanoparticles exhibit a transition temperature from paramagnetism to diamagnetism at a specific temperature, which ranges from 220 K for NiTe_1.15_ to 58 K for NiTe_2_ [6]. The room-temperature magnetization curves for the samples milled for 8 h demonstrate ferromagnetic behavior (Figure 7), which is consistent with results published by Wan et al. [29]. The measured intrinsic coercivity (*H*_c_) values for NiTe, NiTe_2_, and Ni_2_Te_3_ are 134, 131, and 121 Oe, while the corresponding remanence values (*M*_r_) are 30.8 × 10^−3^, 35.9 × 10^−3^ and 5.1 × 10^−3^ emu/g. It has been reported in earlier studies that the magnetic behavior of nickel tellurides depends on the method of synthesis [23]. The mechanochemical method seems to have an advantage when it comes to magnetic properties of as-prepared nickel tellurides since the ferromagnetic properties enable their manipulation through an external magnetic field [51], so they can easily be recycled and reused, making their applications environmentally friendly.

**Figure 8:**
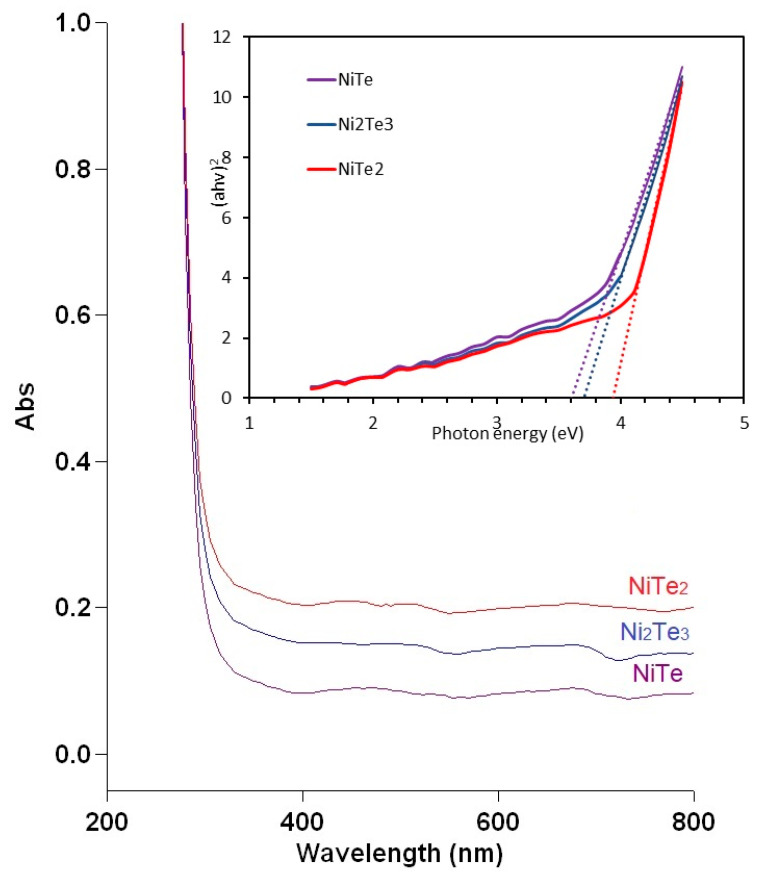
UV-VIS spectra of different nickel tellurides: NiTe, NiTe_2_, and Ni_2_Te_3_ prepared by solvent-free mechanochemical synthesis. The inset represents the Tauc plots used for the determination of the band gap.

The UV-VIS spectra of nickel tellurides are presented in Figure 8. From those data, optical band gap energies were determined using Tauc plots and the equation:

(*αhν*)*^n^* = *A*(*hν* − *E*_g_)

Here, *α* is the absorption coefficient (cm^−1^), *hν* is the photon energy (eV), *A* is a constant, and *n* = 2 for a direct transition. By plotting (*αhν*)*^2^* vs. (*hν*) and extrapolating the linear portion of the curve to the *x*-axis, the band gap value *E*_g_ (eV) can be obtained as the intercept [52]. The band gap energies calculated for NiTe, NiTe_2_, and Ni_2_Te_3_ and are 3.59, 3.94, and 3.70 eV, respectively. The value obtained for NiTe is in very good agreement with the result 3.56 ± 0.01 eV reported in the literature [7] for nanosized NiTe prepared by the reduction method. The results are of considerable interest in the view of further applications of mechanochemically prepared nickel tellurides. One particularly promising area seems to be the use of nickel tellurides as photo-electrocatalysts and photocatalysts for overall water splitting [53,54], making those materials attractive choices as wide band gap semiconductors.

**Figure 9:**
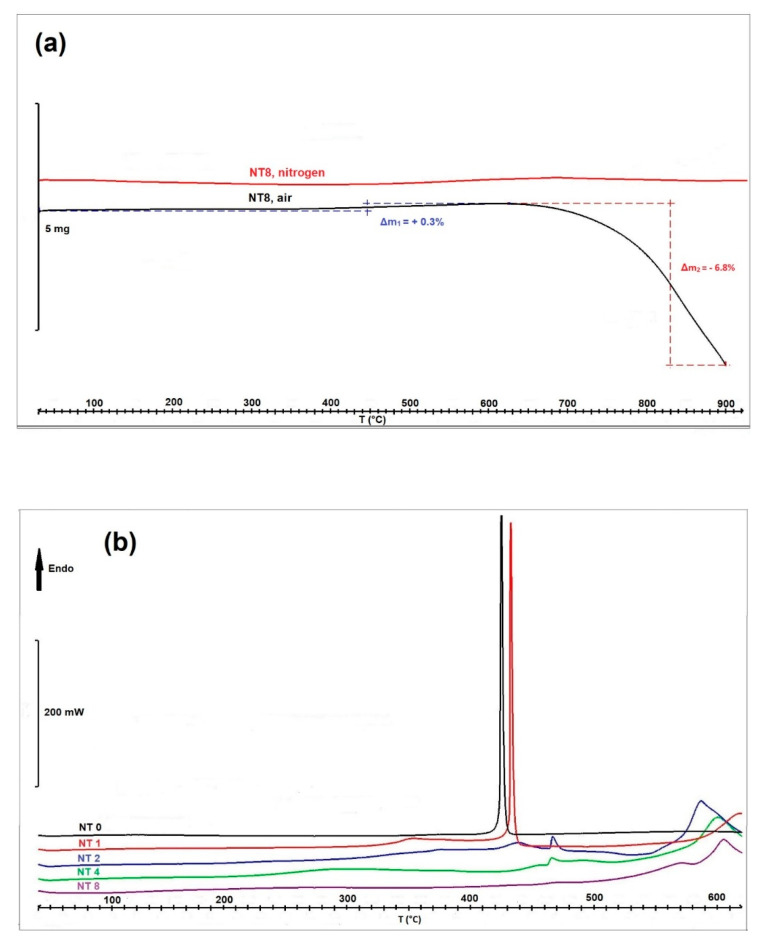
TGA curves of mechanochemically prepared NiTe in nitrogen and air (**a**) and DSC curves of NiTe samples after different milling times measured in nitrogen (**b**).

The results of the thermal analysis of NiTe samples are presented in Figure 9a,b. Figure 9a shows the curves obtained by TGA measurements of samples obtained after 8 h milling time, in nitrogen and in air. Both samples can be regarded as thermally stable up to 450 °C. The sample measured in nitrogen remained stable even at higher temperatures, while the behavior in the air is significantly different: after a negligible weight gain of 0.3% between 450 and 620 °C, the mass drops by 6.8% by further heating up to 900 °C, most probably due to oxidation processes.

The DSC curves, presented in Figure 9b, further confirm the results of XRD measurements, shown in Figure 1. The uppermost curve (NT 0) represents a mixture of Ni and Te (molar ratio 1:1) while the other curves correspond to products obtained by mechanical milling of the 1:1 mixture for 1 h, 2 h, 4 h, and 8 h. The sharp peaks in the NT 0 and NT 1 samples correspond to the melting point of tellurium (449.5 °C), indicating the presence of unreacted Te after only 1 h milling time. After 2 h milling time, the Te melting peak is still noticeable, while after 4 h and 8 h, no Te melting peak can be observed, indicating that Te has successfully reacted with Ni to form NiTe, which is in good agreement with results obtained by XRD.

## 4. Conclusions

Nickel tellurides with different compositions, namely, NiTe, NiTe_2_, and Ni_2_Te_3_, have been synthesized from elemental precursors using the mechanochemical method. Powder XRD measurements reveal that pure products without detectable amounts of precursors were obtained after milling times of 8–12 h. A detailed morphological study by SEM, TEM, and DLS measurements revealed the structure consisting of agglomerates in submicron to micron sizes, while nanosized primary particles can be observed at higher magnification. The method is simple and environmental-friendly by avoiding the use of solvents, toxic precursors, and energy-consuming reaction conditions. The tendency to form clusters, which might be considered one of the drawbacks of the mechanochemical method, could be overcome during further research by using surfactants during the milling process, preventing agglomeration during the synthesis.

## References

[1] Li B., Xie Y., Huang J., Su H., Qian Y. (1999). Solvothermal synthesis to NiE_2_ (E = Se, Te) nanorods at low temperature. Nanostruct. Mater..

[2] Kristl M., Dojer B., Gyergyek S., Kristl J. (2017). Synthesis of nickel and cobalt sulfide nanoparticles using a low cost sonochemical method. Heliyon.

[3] Kristl M., Gyergyek S., Kristl J. (2018). Nanostructured nickel sulfides with different stoichiometries prepared by mechanochemical synthesis. Chalcogenide Lett..

[4] Zhang H.T., Xiong Y.M., Luo X.G., Wang C.H., Li S.Y., Chen X.C. (2002). Hydrothermal synthesis and characterization of NiTe alloy nanocrystallites. J. Cryst. Growth.

[5] Arvhult C.M., Guéneau C., Gossé S., Selleby M. (2019). Thermodynamic assessment of the Ni-Te system. J. Mater. Sci..

[6] Lei Y.X., Zhou J.P., Wang J.Z., Miao N.X., Guo Z.Q., Hassan Q.U. (2017). Novel magnetic properties of uniform NiTe_x_ nanorods selectively synthesized by hydrothermal method. Mater. Des..

[7] Pradhan S., Das R., Biswas S., Das D.K., Bhar R., Bandyopadhyay R., Pramanik P. (2017). Chemical synthesis of nanoparticles of nickel telluride and cobalt telluride and its electrochemical applications for determination of uric acid and adenine. Electrochim. Acta.

[8] Amin B.G., de Silva U., Masud J., Nath M. (2019). Ultrasensitive and Highly Selective Ni_3_Te_2_ as a Nonenzymatic Glucose Sensor at Extremely Low Working Potential. ACS Omega.

[9] Yu Z.J., Jiao S.Q., Tu J.G., Luo Y.W., Song W.L., Jiao H.D., Wang M.Y., Chen H.S., Fang D.N. (2020). Rechargeable Nickel Telluride/Aluminum Batteries with High Capacity and Enhanced Cycling Performance. ACS Nano.

[10] Zhou P., Fan L., Wu J., Gong C., Zhang J., Tu Y. (2016). Facile hydrothermal synthesis of NiTe and its application as positive electrode material for asymmetric supercapacitor. J. Alloys Compd..

[11] Manikandan M., Subramani K., Sathish M., Dhanuskodi S. (2018). NiTe Nanorods as Electrode Material for High Performance Supercapacitor Applications. ChemistrySelect.

[12] Ye B., Huang M., Fan L., Lin J., Wu J. (2019). Co ions doped NiTe electrode material for asymmetric supercapacitor application. J. Alloys Compd..

[13] Anand T.J.S., Zaidan M. (2014). Electro synthesized NiTe_2_ Thin Films with the Influence of Additives. Adv. Mater. Res..

[14] Chia X., Sofer Z., Luxa J., Pumera M. (2017). Unconventionally Layered CoTe_2_ and NiTe_2_ as Elactrocatalysts for Hydrogen Evolution. Chem. Eur. J..

[15] Bhat K.S., Barshilia H.C., Nagaraja H.S. (2017). Porous nickel telluride nanostructures as bifunctional electrocatalysts towards hydrogen and oxygen evolution reaction. Int. J. Hydrogen Energy.

[16] Oh J., Park H.J., Bala A., Kim H.S., Liu N., Choo S., Lee M.H., Kim S.J., Kim S. (2020). Nickel telluride vertically aligned thin film by radio-frequency magnetron sputtering for hydrogen evolution reaction. APL Mater..

[17] de Silva U., See J., Liyanage W.P.R., Masud J., Wu J.P., Yang W.L., Chen W.T., Prendergast D., Nath M. (2021). Understanding the Structural Evolution of a Nickel Chalcogenide Electrocatalyst Surface for Water Oxidation. Energy Fuels.

[18] Anantharaj S., Karthick K., Kundu S. (2018). NiTe_2_ nanowire Outperforms Pt/C in High-Rate Hydrogen Evolution at Extreme pH Conditions. Inorg. Chem..

[19] Wang Z.C., Zhang L.X. (2018). Nickel Ditelluride nanosheet Arrays: A Highly Efficient Electrocatalyst for the Oxygen Evolution Reaction. ChemElectroChem.

[20] Wang Z., Guo P., Liu M., Guo C., Liu H., Wei S., Zhang J., Lu X. (2019). Rational Design of Metallic NiTe*_x_* (*x* = 1 or 2) as Bifunctional Electrocatalysts for Efficient Urea Conversion. ACS Appl. Energy Mater..

[21] Wang Z., Zhang L. (2018). In situ growth of NiTe nanosheet film on nickel foam as electrocatalysts for oxygen evolution reaction. Elecrochem. Commun..

[22] Gulay L.D., Olekseyuk I.D. (2004). Crystal structures of the compounds Ni_3_Te_2_, Ni_3-δ_Te_2_ (δ = 0.12) and Ni_1.29_Te. J. Alloys Compd..

[23] Umeyama N., Tokumoto M., Yagi S., Tomura M., Tokiwa K., Fujii T., Toda R., Miyakawa N., Ikeda S.I. (2012). Synthesis and Magnetic Properties of NiSe, NiTe, CoSe, and CoTe. Jpn. J. Appl. Phys..

[24] Parkin I.P. (1996). Solid state metathesis reaction for metal borides, silicides, pnictides and chalcogenides: Ionic or elemental pathways. Chem. Soc. Rev..

[25] Brennan J.G., Siegrist T., Stuczynski S.M., Steigerwald M.L. (1989). The transition from molecules to solids-molecular syntheses of Ni_9_Te_6_(PEt_3_)_8_, Ni_20_Te_18_(PEt_3_)_12_, and NiTe. J. Am. Chem. Soc..

[26] Mu Y.N., Li Q., Lv P., Chen Y.L., Ding D., Su S., Zhou L.Y., Fu W.Y., Yang H.B. (2014). Fabrication of NiTe films by transformed electrodeposited Te thin films on Ni foils and their electrical properties. RSC Adv..

[27] Zhao B., Dang W.Q., Liu Y., Li B., Li J., Luo J., Zhang Z.W., Wu R.X., Ma H.F., Sun G.Z. (2018). Synthetic Control of Two-Dimensional NiTe_2_ Single Crystals with Highly Uniform Thickness Distributions. J. Am. Chem. Soc..

[28] Peng Q., Dong Y., Li Y. (2003). Synthesis of Uniform CoTe and NiTe Semiconductor Nanocluster Wires through a Novel Coreduction Method. Inorg. Chem..

[29] Wan L.J., Liu J.H., Huang X.J. (2014). Novel magnetic nickel telluride nanowires decorated with thorns: Synthesis and their intrinsic peroxidase-like activity for detection of glucose. Chem. Commun..

[30] Liu X.Y., Hu R.Z., Chai L.L., Li H.B., Gu J., Qian Y.T. (2009). Synthesis and Characterization of Hexagonal NiTe_2_ Nanoplates. J. Nanosci. Nanotechnol..

[31] Jiang L., Zhu Y.J., Cui J.B. (2010). Cetyltrimethylammonium bromide assisted self-assembly of NiTe_2_ nanoflakes: Nanoflake arrays and their photoluminescence properties. J. Solid State Chem..

[32] Suryanarayana C. (2001). Mechanical alloying and milling. Prog. Mater. Sci..

[33] Zhang D.L. (2004). Processing of advanced materials using high-energy mechanical milling. Prog. Mater. Sci..

[34] Matyja E., Prusik K., Zubko M., Dercz G., Glowka K. (2019). Structure of the Ni-Co-Mn-In alloy obtained by mechanical alloying and sintering. J. Alloys Compd..

[35] Gomez-Lopez P., Espro C., Rodriguez-Padron D., Balu A.M., Ivars-Barcelo F., Moreda O.I., Alvarado-Beltran C.G., Luque R. (2021). Mechanochemical Preparation of Magnetically Separable Fe and Cu-Based Bimetallic Nanocatalysts for Vanilin Production. Nanomaterials.

[36] de Brito Neto F.M., Takeno M.L., da Costa Pinto C., Triches D.M., Manzato L., de Souza S.M. (2019). Structural and thermal studies of SmNbO_4_ polymorphs produced by mechanical alloying. Mater. Lett..

[37] Garcia-Garcia F.J., Sayagues M.J., Gotor F.J. (2021). A Novel, Simple and Highly Efficient Route to Obtain PrBaMn_2_O_5+δ_ Double Perovskite: Mechanochemical Synthesis. Nanomaterials.

[38] Hredzak S., Brinacin J., Sepelak V. (2019). Mechanochemically Synthesized Coal-Based magnetic Carbon Composites for Removing As(V) and Cd(II) from Aqueous Solutions. Nanomaterials.

[39] Olszewski R., Nadolska M., Lapinski M., Przesniak-Welenc M., Cieslik B.M., Zelechowska K. (2019). Solvent-Free Synthesis of Phosphonic Graphene Derivative and Its Application in Mercury Ions Adsorption. Nanomaterials.

[40] Zhang B., Wang H.W., Liu C., Li D.J., Kim H.K., Harris C., Lao C.Y., Abdelkader A., Xi K. (2019). Facile mechanochemical synthesis of non-stoichiometric silica-carbon composite for enhanced lithium storage properties. J. Alloy. Compd..

[41] Kristl M., Ban I., Gyergyek S. (2013). Preparation of Nanosized Copper and Cadmium Chalcogenides by Mechanochemical Synthesis. Mater. Manuf. Process..

[42] Kristl M., Gyergyek S., Srt N., Ban I. (2016). Mechanochemical Route for the Preparation of Nanosized Aluminium and Gallium Sulfide and Selenide. Mater. Manuf. Process..

[43] Weller D.P., Morelli D.T. (2017). Rapid synthesis of zinc and nickel co-doped tetrahedrite thermoelectrics by reactive spark plasma sintering and mechanical alloying. J. Alloys Compd..

[44] Falkenbach O., Loeh M.O., Wiegand C.W., Schmitz A., Hartung D., Koch G., Klar P.J., Mueller E., Schlecht S. (2017). Structural and Thermoelectric Properties of Nanostructured Nominally Stoichiometric Pb_1-x_Bi_x_Te Prepared by Mechanical Alloying. J. Electron. Mater..

[45] Hu P.F., Xie C., Mao Z.H., Liang X. (2019). A Mechanochemical Route for ZnS Nanocrystals, and Batch Sorting along Size Distribution. Nanomaterials.

[46] Zhang X.F., Sun Q.X., Zheng M.X., Duan Z.H., Wang Y.H. (2020). Solar Sell Applications of Solution-Processed AgInGaSe_2_ Thin Films and Improved Properties by Sodium Doping. Nanomaterials.

[47] Campos C.E.M. (2014). Solid state synthesis and characterization of NiTe nanocrystals. J. Nano Res..

[48] Unni G.E., Sasi S., Nair A.S. (2016). Higher open-circuit voltage set by cobalt redox shuttle in SnO_2_ nanofibers-sensitized CdTe quantum dots solar cells. J. Energy Chem..

[49] Larichev Y.V. (2021). Application of DLS for metal nanoparticle size determination in supported catalysts. Chem. Pap..

[50] Ban I., Markuš S., Gyergyek S., Drofenik M., Korenak J., Helix-Nielsen C., Petrinić I. (2019). Synthesis of Poly-Sodium_Acrylate (PSA)-Coated Magnetic nanoparticles for Use in Forward Osmosis Draw Solutions. Nanomaterials.

[51] Stergar J., Maver U., Bele M., Gradišnik L., Kristl M., Ban I. (2020). NiCu-silica nanoparticles as a potential drug delivery system. J. Sol. Gel Sci. Technol..

[52] Shinde P.S., Go G.H., Lee W.J. (2012). Facile growth of hierarchical hematite (α-Fe_2_O_3_) nanopetals on FTO by pulse reverse electrodeposition for photoelectrochemical water splitting. J. Mater. Chem..

[53] Sadaquat M., Manzoor S., Nisar L., Hassan A., Tyagi D., Shah J.H., Ashiq M.N., Joya K.S., Alshahrani T., Najam-ul-Haq M. (2021). Iron doped nickel ditelluride hierarchical nanoflakes array directly grown on nickel foam as robust electrodes for oxygen evolution reaction. Electrochim. Acta.

[54] Woods-Robinson R., Han Y., Zhang H., Ablekim H., Khan I., Persson K.A., Zakutayev A. (2020). Wide Band Gap Chalcogenide Semiconductors. Chem. Rev..

